# Tuning the Properties of Active Microtubule Networks by Depletion Forces

**DOI:** 10.1021/acs.langmuir.1c00426

**Published:** 2021-06-16

**Authors:** Vahid Nasirimarekani, Tobias Strübing, Andrej Vilfan, Isabella Guido

**Affiliations:** † University of the Basque Country UPV/EHU, 01006 Vitoria-Gasteiz, Spain; ‡ Max Planck Institute for Dynamics and Self-Organization, 37077 Göttingen, Germany; § Jožef Stefan Institute, 1000 Ljubljana, Slovenia

## Abstract

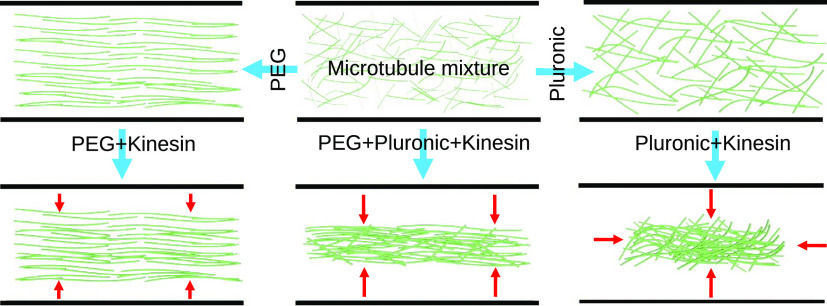

Suspensions of microtubules and nonadsorbing particles form thick and long bundles due to depletion forces. Such interactions act at the nanometer scale and define the structural and dynamical properties of the resulting networks. In this study, we analyze the depletion forces exerted by two types of nonadsorbing particles, namely, the polymer, poly(ethylene glycol) (PEG), and the block copolymer, Pluronic. We characterize their effects both in passive and active networks by adding motor proteins to the suspensions. By exploiting its bundling effect via entropic forces, we observed that PEG generates a network with thick structures showing a nematic order and larger mesh size. On the other hand, Pluronic builds up a much denser gel-like network without a recognizable mesh structure. This difference is also reflected in the network activity. PEG networks show moderate contraction in lateral directions while Pluronic networks exhibit faster and isotropic contraction. Interestingly, by mixing the two nonadsorbing polymers in different ratios, we observed that the system showed a behavior that exhibited properties of both agents, leading to a robust and fast responsive structure compared to the single-depletant networks. In conclusion, we show how passive osmotic compression modifies the distribution of biopolymers. Its combination with active motors results in a new active material with potential for nanotechnological applications.

## Introduction

The collective behavior of molecules transducing energy into motion underlies many biological processes and enables vital functions that lead to what we call “life”. Interest in exploring patterns, dynamics, and functionalities that these intracellular microscopic elements can generate has tremendously grown in the last decades. Moreover, their potential for the development of new bioinspired nanomaterials is currently providing the impetus for new research directions.^1^ Bottom-up assembled biomolecular structures have started to emerge as model systems that can reproduce the behavior of a living ensemble and elucidate fundamental mechanisms underlying emergent behavior in nature. Convenient biological components for such active materials are the biopolymers and motor proteins from the cellular cytoskeleton. In the cell, these components form self-organizing networks with different architectures according to the needs for spatial and functional organization. Early foundational studies showed that simplified *in vitro* systems greatly improved our understanding of the complex self-organizing organisms they originate from.^2−6^

Microtubules are one class of these biopolymers. They are constituted of α and β tubulin subunits that first form dimers, which then polymerize into long, semiflexible tubular structures with a diameter of 25 nm. As shown in previous studies,^2,3^ the properties of the networks they form *in vitro* can be influenced by adding force-generating components, such as kinesin-1 motor proteins that cross-link the polymerized and stabilized microtubules and produce active stress by converting energy from adenosine triphosphate (ATP) hydrolysis into motion.

**Figure 1:**
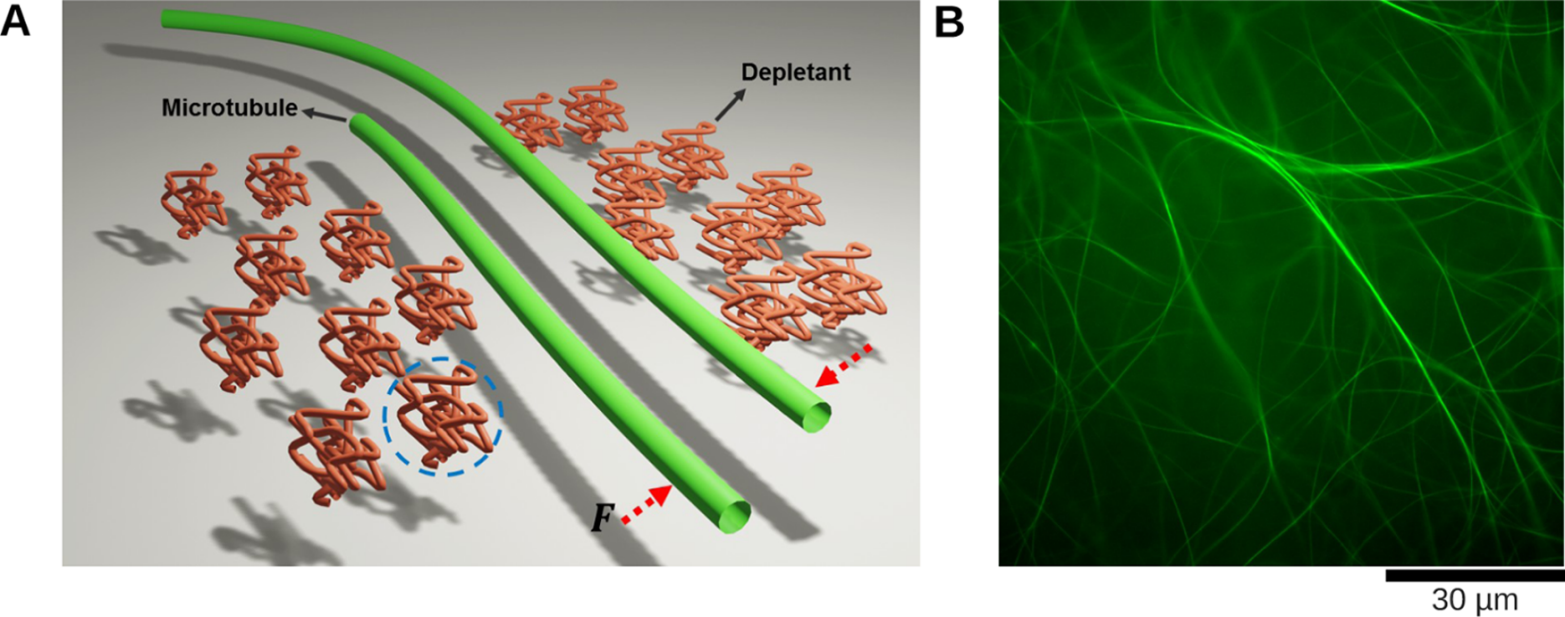
Bundling of microtubules by depletion of nonabsorbing poly(ethylene glycol) (PEG) molecules. (A) Schematic representation of the depletion effect of PEG on microtubules and attraction force (*F*) bundling them. (B) Micrograph of thick microtubule bundles formed due to 1% PEG in a solution.

An additional way to tune the features of these materials can be obtained by adding passive crowding agents such as nonionic polymers, also known as depletants, that induce attractive interactions between rod-like particles of different natures and bundle them.^7−15^ The attractive process between the filaments due to the added depletant is known as depletion interaction and is driven by entropic forces,^7^ i.e., the depletant macromolecules tend to maximize the total volume available to them for increasing the entropy. When mixed with microtubules and motor proteins, the way to reduce the excluded volume is by bundling the filaments. Consequently, the spacing between microtubules in the bundles is reduced to a few nanometers and this arrangement provides the ideal distance for the motors to increase their binding probability^16^ (Figure 1). The motor proteins cross-link the filaments and let them slide against each other, exerting both contractile and extensile forces in the network.^17−21^ Thus, the system results in an active interconnected network, a nanomaterial characterized by an architecture that influences its geometrical and mechanical properties.^22^

In this study, we show how depletion interactions caused by polymers of different natures in solution and activity of motor proteins differently affect the properties and dynamic behavior of biopolymer networks. For this purpose, we used two nonionic depletion agents, namely, the polymer, poly(ethylene glycol) (PEG), and the block copolymers, poloxamers, known under the trade name Pluronic F127 (hereinafter referred to as Pluronic). PEG is a very common and widely used linear polymer, known to create effective attraction between protein molecules of different natures due to depletion forces and widely used to bundle microtubules.^23−26^ Pluronic is an amphiphilic triblock copolymer with a central hydrophobic chain of polyoxypropylene (poly(propylene oxide)) and two hydrophilic chains of PEG.^27,28^ At low temperatures and/or concentrations, Pluronic exists in solution as individual coils (unimers). By increasing the copolymer concentration and/or solution temperature, it forms stable micelles, which have a hydrodynamic radius 1 order of magnitude larger than the radius of gyration of the unimers.^29,30^ In this study, after a short preparation time at 37 °C, the experiments were performed at room temperature and the depletant concentration was set to 1%.

In addition to confirming the capability of the two depletants to bundle biopolymers, we observed that they generate networks with different properties when they are mixed with microtubules and are confined in a microfluidic channel. By combining the depletion effect with the activity of motor proteins, we show how the network architecture modulates the forces that the motors exert on the filaments. Moreover, we show their cooperative effect on the active network when the two depletant agents are mixed together. We observed a resultant new material with properties simultaneously dependent on PEG and Pluronic.

## Materials and Methods

### Microfluidic Channel and Nonadsorbing Surface Coatings

**Figure 2:**
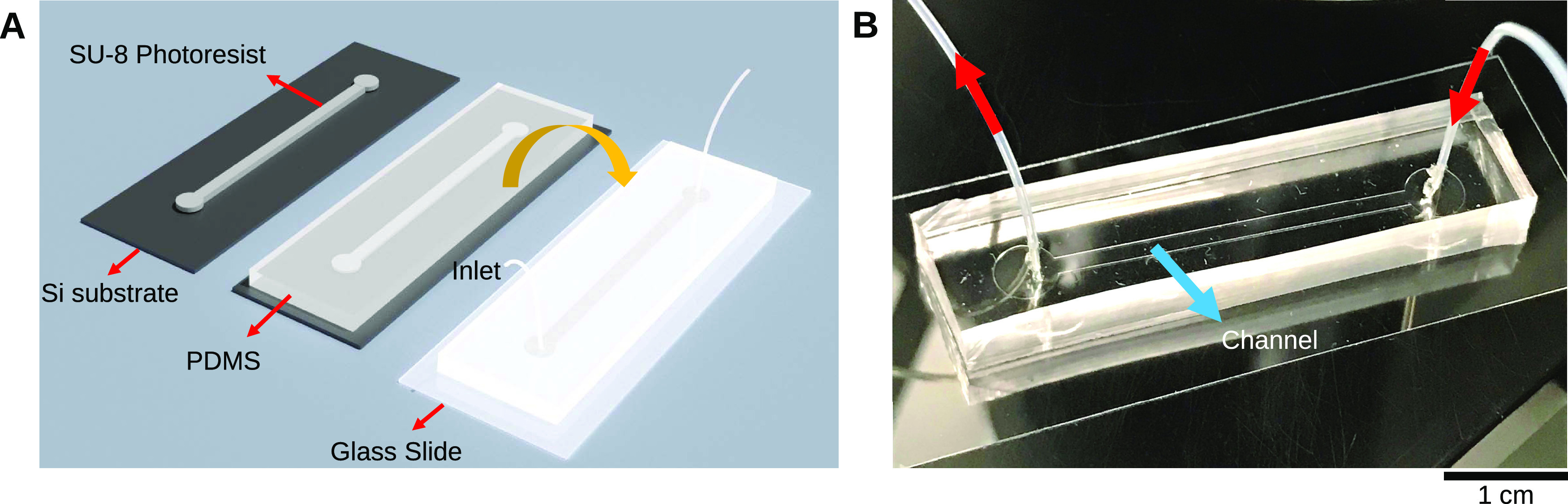
Experimental microfluidic channel. (A) Schematic representation of the PDMS device production by soft lithography. (B) Experimental channel with inlet and outlet tubings for injection of the sample mixture. After injection, the tubings were removed and the channel was sealed using vacuum grease.

An experimental chamber was created on a microfluidic device, which was obtained by molding polydimethylsiloxane (PDMS, a 10:1 mixture with a curing agent, Sylgard 184, Dow Corning Europe SA) on SU-8 wafers. The microfluidic channel was 1.5 mm wide, 100 μm high, and 30 mm long. Inlets and outlets for the active mixture were punched through the PDMS using a syringe tip. Plasma activation was applied on PDMS and a coverslip (64 × 22 mm, VWR) to bond them to close the channel (Figure 2). Coverslips were cleaned by washing with 100% ethanol and rinsing in deionized water. They were further sonicated in acetone for 30 min and incubated in ethanol for 10 min at room temperature. This was followed by incubation in a 2% Hellmanex III solution (Hellma Analytics) for 2 h, extensive washing in deionized water, and drying with a filtered airflow. The cleaned coverslips were immediately activated in oxygen plasma (FEMTO, Diener Electronics, Germany) for 30 s at 0.5 mbar and sealed to the PDMS. Poly(l-lysine)-*graft*-poly(ethylene glycol) (PLL-*g*-PEG) (SuSoS AG, Switzerland) at a final concentration of 0.1 mg/mL in 10 mM 4-(2-hydroxyethyl)-1-piperazineethanesulfonic acid (HEPES) (pH 7.4, at room temperature) was injected into the channel and incubated for 1 h. Finally, M2B buffer (80 mM PIPES, adjusted to pH = 6.9 with KOH, 1 mM EGTA, 2 mM MgCl_2_) was used to remove the excess PLL-*g*-PEG from the channel. The input and output of the channel were sealed using vacuum grease after the injection of the mixture.

### Microtubule Active Mixture

For the preparation of the sample, we mainly followed a protocol available in the literature.^16^ A microtubule mixture was prepared by 2.7 mg/mL 488 HiLyte-labeled porcine brain tubulin (Cytoskeleton, Inc.) in M2B with 5 mM MgCl_2_, 1 mM GTP, 5% dimethyl sulfoxide (DMSO), 0.5 mg/mL glucose, 0.65 mM dithiothreitol (DTT), 0.2 mg/mL glucose oxidase (Sigma G2133), 0.05 mg/mL catalase (Sigma C40), and 2.4 mM Trolox (Sigma 238813).

For the active network experiments, kinesin 401 clusters were prepared. The plasmid that codes biotin-labeled kinesin 401 (K401) was a gift from Jeff Gelles (pWC2—Addgene plasmid #15960; http://n2t.net/addgene:15960; RRID Addgene 15960).^31^ Kinesin 401 was purified as previously published^32,33^ and kinesin–streptavidin clusters were prepared by mixing 0.2 mg/mL kinesin 401, 0.9 mM DTT, and 0.1 mg/mL streptavidin (Invitrogen, S-888) dissolved in M2B and incubated on ice for 15 min. A total of 4 μL of this mixture was mixed with ATP at a final concentration of 1 mM, 1.7 μL of pyruvate kinase/lactic dehydrogenase (PK/LDH, Sigma, P-0294), 32 mM phosphoenol pyruvate (PEP, VWR AAB20358-06), 8 mM MgCl_2_, and 7 μM taxol for microtubule stabilization to form a solution of active clusters that was added to the microtubule solution described above. Overall, 1% PEG or Pluronic was added^21^ to this resultant mixture. In the passive case experiments, kinesin and ATP were replaced by M2B buffer. The microtubules and the active cluster solution were mixed 15 min before the onset of the incubation for polymerization, incubated at 37 °C for 30 min, and afterward introduced into the channel using a syringe.

### Microscopy and Image Analysis

Image acquisition was performed using an inverted fluorescence microscope Olympus IX-71 with a 4× objective (Olympus, Japan) or a 60× oil-immersion objective (Olympus, Japan), depending on the experimental setup. For excitation, a Lumen 200 metal arc lamp (Prior Scientific Instruments.) was applied. The images were recorded with a CCD camera (CoolSnap HQ2, Photometrics). The frames were acquired with a variable rate according to the experiment with an exposure time of 500 ms for a variable time according to the experiment. ImageJ software was used for the analysis of the acquired images.

## Results and Discussion

We conducted experiments by mixing taxol-stabilized microtubules with the two different depletants, PEG and Pluronic, and we studied their effects on the network behavior in three different cases: (i) a nonactive network made of a depletant (PEG or Pluronic) and biopolymers, (ii) a network, made of a depletant, biopolymers and motor proteins, and (iii) an active mixed case, containing both depletants, biopolymers, and motor proteins. In the following sections, we characterize and compare the resultant networks.

### Depletion by PEG and Pluronic in a Nonactive Network

**Figure 3:**
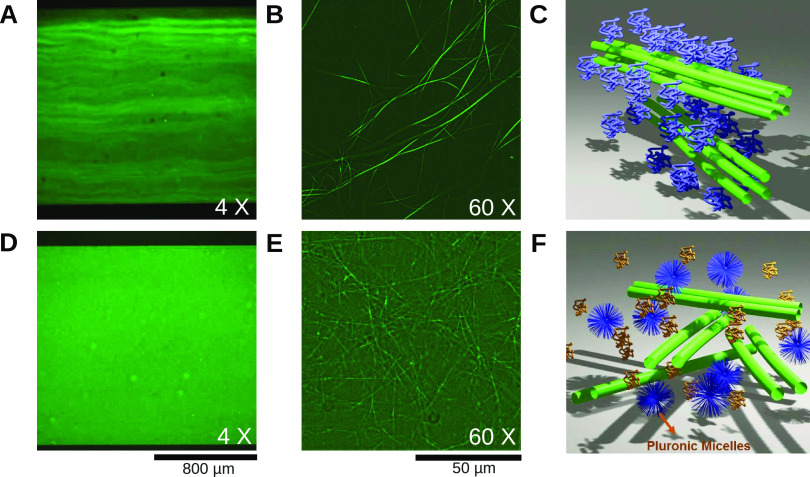
Comparison between microtubule bundles resulting from depletion forces induced by PEG and Pluronic in a nonactive case in a microfluidic channel. (A) PEG-induced depletion forces caused longer and thicker bundles, which were aligned along the channel. (B) Micrograph (60×) of microtubule bundles. (C) Schematics of bundle formation and spatial organization with PEG. (D) In contrast to the PEG network, the structure of the Pluronic network is not visible at a 4× magnification, as the system appears like a compact gel. (E) Micrograph (60×) confirms formation of shorter and thinner bundles with a random spatial distribution in the presence of Pluronic. (F) Schematics of the depletion effect by Pluronic as a mixture of micelles and single unimers.

Taxol-stabilized microtubules were mixed with a depletant (either PEG or Pluronic) at a final concentration (w/v) of 1% and introduced into the microfluidic channel. The dynamics of the formed networks was recorded from the onset of the experiment (Figure 3). In this nonactive system, the result of the interactions between the depletant and biopolymers is a network with properties that depend on the nature of the depletant. Figure 3 shows a comparison between the networks resulting from the addition of PEG (Figure 3A–C) or Pluronic (Figure 3D–F). As observed in our previous study,^21^ the depletion effect due to PEG resulted in the formation of a network made of visible thick and long bundles that were aligned along the longitudinal axis of the channel and formed a cross-linked network (Figure 3A). We can denote this arrangement as nematic, which usually refers to rod-shaped filaments like the microtubules that are organized parallel or antiparallel to each other along a preferred axis, forming a liquid crystalline phase. On the other hand, the addition of Pluronic resulted in an isotropic distribution of filaments inside the channel with no recognizable mesh structure (Figure 3D). Thus, the bundles had no preferred orientation to each other and pointed to all spatial directions with a homogeneous distribution of probabilities. This was made more clear by visualizing the networks at a higher magnification (60×), where we could observe the difference in the structure formation and spatial distribution of PEG and Pluronic networks (Figure 3B,E). While the induced attraction between the microtubules and the minimization of the excluded volume by PEG caused a distinct phase separation between the bundles and the PEG macromolecules, the biopolymer bundles due to Pluronic appeared thinner, more numerous, more cross-linked, and isotropically distributed in space. This might explain why the Pluronic network showed a gel-like behavior, while the PEG one was more porous and diluted.

This different behavior may be due to the different chemical structures of the two depletants and the corresponding depletion effects that they cause in the bulk of a microtubule mixture. Both PEG and Pluronic are nonionic and nonabsorbing structures and do not interact with microtubules. Furthermore, in both cases, PEG molecules tend to maximize the volume available to minimize the free energy of the system at equilibrium.^7^ When the distance between microtubules is smaller than the diameter of PEG molecules (radius of gyration of PEG is 7 nm), no PEG molecule can invade this interstitial space. Thus, an osmotic pressure of the PEG molecule is generated and an equivalent attractive force acts on the microtubules that are bundled as a consequence.^7^ The thickness of the bundles and the spatial organization of the biopolymers inside them depend on the molecular weight and concentration of PEG in a solution, which determine the osmotic pressure that brings the biopolymers together.^34^ On the other hand, the Pluronic concentration of 1% used in our experiments is slightly above its critical micelle concentration (CMC), which is 0.7% at 25 °C.^35^ Thus, the depletion force was generated by a mixture of Pluronic unimers and micelles, which have a bigger dimension (hydrodynamic radius around 20 nm, much bigger than the radius of gyration of the unimers^29,30^). Hence, micelles reduced the concentration of unimers in solution and consequently the osmotic pressure of the system on the microtubules. The resultant network had thinner bundles that were isotropically distributed and highly cross-linked. A schematic representation of this effect is shown in Figure 3C,F.

**Figure 4:**
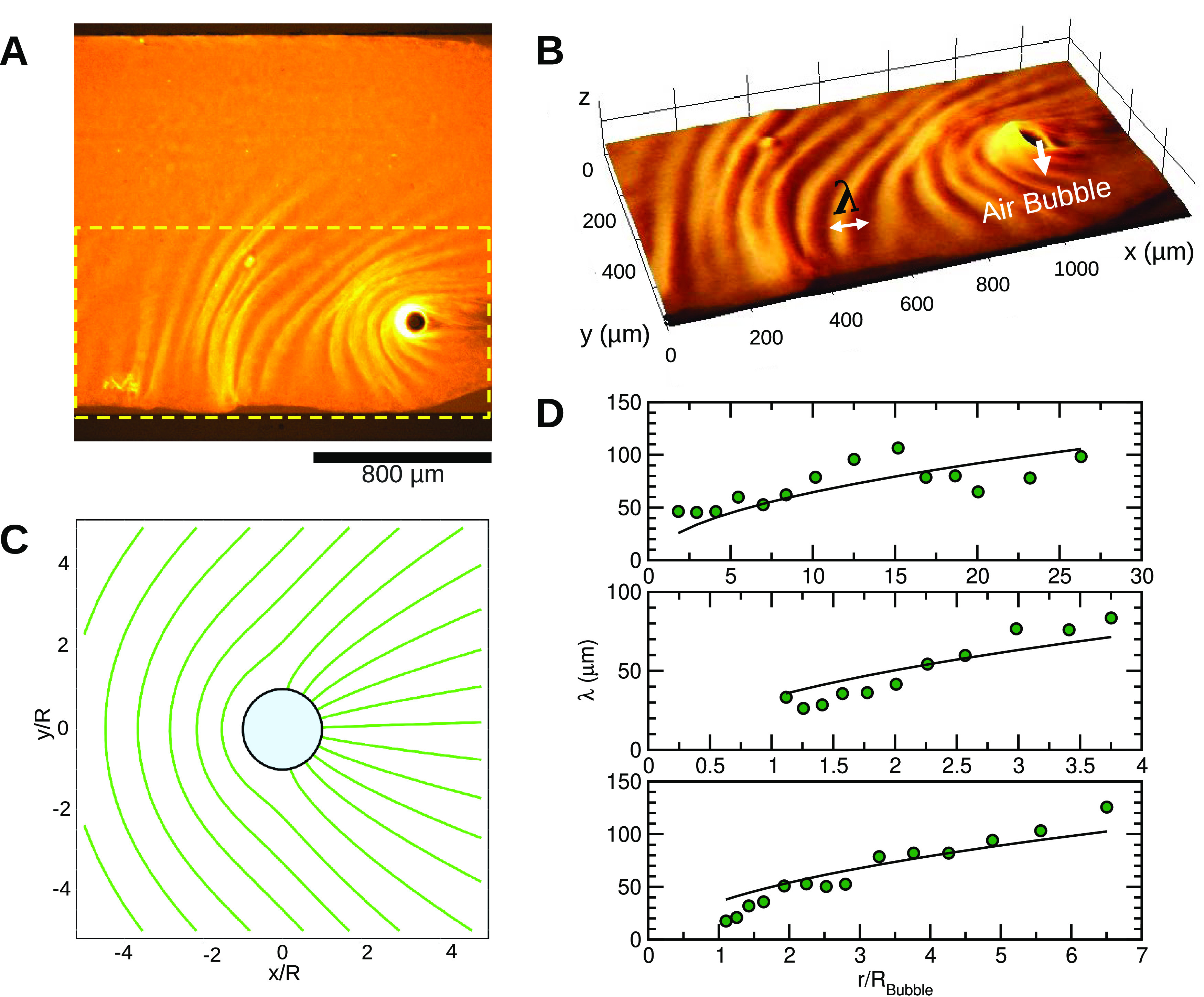
Wrinkling pattern in a network with Pluronic formed around an air bubble. (A) Microtubule network wrinkled around an air bubble like a compact elastic sheet. (B) Inset of wrinkles and their wavelength measurement. (C) Theoretically calculated stress field around a bubble (blue) in an elastic medium. The green lines indicate directions normal to the direction of maximal compressive stress. The predicted stress direction shows striking similarity with the observed direction of wrinkles. (D) Wrinkle wavelength as a function of the distance from the center of the bubble for three different samples. The wavelengths are fitted with a √*r* function as predicted by the elastic theory.

The gel-like behavior of the Pluronic network was clearly observable in the presence of obstacles that hindered its movement inside the channel (Figure 4). Due to the accidental but still common presence of air bubbles trapped in the channel when injecting the mixture, the Pluronic network formed regular wrinkles (Figure 4A,B) when displaced against the obstacle. This behavior can be understood with a theoretical model that treats the network as an ideal two-dimensional (2D) compressible elastic material and the bubble as a circular hole with zero shear stress on its perimeter. As the network moves relative to the bubble, a stress field arises around it. As a consequence, wrinkles in the direction of the compressive stress appear. An exact solution to the problem is derived in the next section. Such a response is not observed in the PEG network, which showed a viscoelastic behavior.

### Elastic Deformations and Wrinkling

To understand the shape of the wrinkling pattern described in the previous section, we calculate the stress field in the sheet caused by a relative displacement of the bubble. We treat the sheet as a 2D elastic plate (i.e., the stresses are calculated in the planar sheet, before out-of-plane deformations appear) and assume that the deformations are small and can be calculated in a linear regime. The deformations of an elastic sheet, described by the displacement **u**(**x**), are governed by the 2D Navier equation
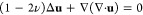
1where *ν* is the Poisson ratio. In the following, we assume an ideally compressible material with *ν* = 0. The Green’s function for the displacement as a response to a point force (*u*_*i*_ = *G*_*ij*_*F*_*j*_) is^36^
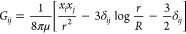
2and is uniquely defined up to an arbitrary constant in the diagonal term. The Green’s function for the stress (σ_*ij*_ = *T*_*ijk*_*F*_*k*_) reads
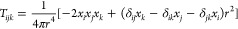
3We now calculate the displacement and stress around a fixed bubble with radius *R* that exerts a force on the surrounding elastic medium that has been displaced. We assume that the bubble shape is stiff, such that the material cannot move radially (**u**(*r* = *R*)·**ê**_*r*_ = 0). Furthermore, the tangential stress on its surface needs to be zero, **ê**_*t*_ ·σ ·**ê**_*r*_ = 0. Here, **x** is defined as the position relative to the center of the bubble, *r* = |**x**|, **ê**_*r*_ = **x**/*r* and **ê**_*t*_ = **ê**_*z*_ × **ê**_*r*_. We construct the solution in a similar way as the flow around a bubble in a zero Reynolds number fluid.^37^ Both boundary conditions are fulfilled with the following superposition of a force and a force quadrupole
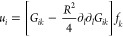
4
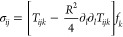
5The force quadrupole terms have the form
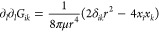
6
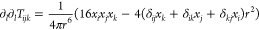
7The wrinkles form in the direction of the compressive stress, such that the wave crests are orthogonal to the eigenvectors corresponding to the smaller eigenvalue of the stress tensor σ_*ij*_ (negative eigenvalues represent compressive stress). A plot showing the calculated directions of waves is shown in Figure 4C, and it reproduces the patterns seen experimentally.

To estimate the wavelength, we follow a similar argumentation as with active nematic sheets, where the wavelength is proportional to , with σ_1_ denoting the smaller eigenvalue of the stress tensor.^21^ In the forward direction (direction of the force), the calculated stress is . The predicted wrinkle wavelength is, therefore,
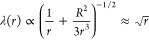
8The relationship is compared to measurements in three samples with different-sized bubbles in Figure 4D, largely confirming the dependence.

### Depletion by PEG and Pluronic in Active Networks

By including molecular motors (kinesin-1), we were able to observe the active behavior of the two networks. Active PEG networks show different kinds of behaviors when kinesin motors exert forces on the microtubules. The combination of depletion attraction and active cross-linking of motors leads to network contraction along the *y*- and *z*-directions. In a certain parameter range, a 3D wrinkling instability emerges, as we showed in a previous study.^21^ While the longitudinal expansion is relatively weak and sensitive to the network conditions, the lateral contraction is a robust feature of an active network.

**Figure 5:**
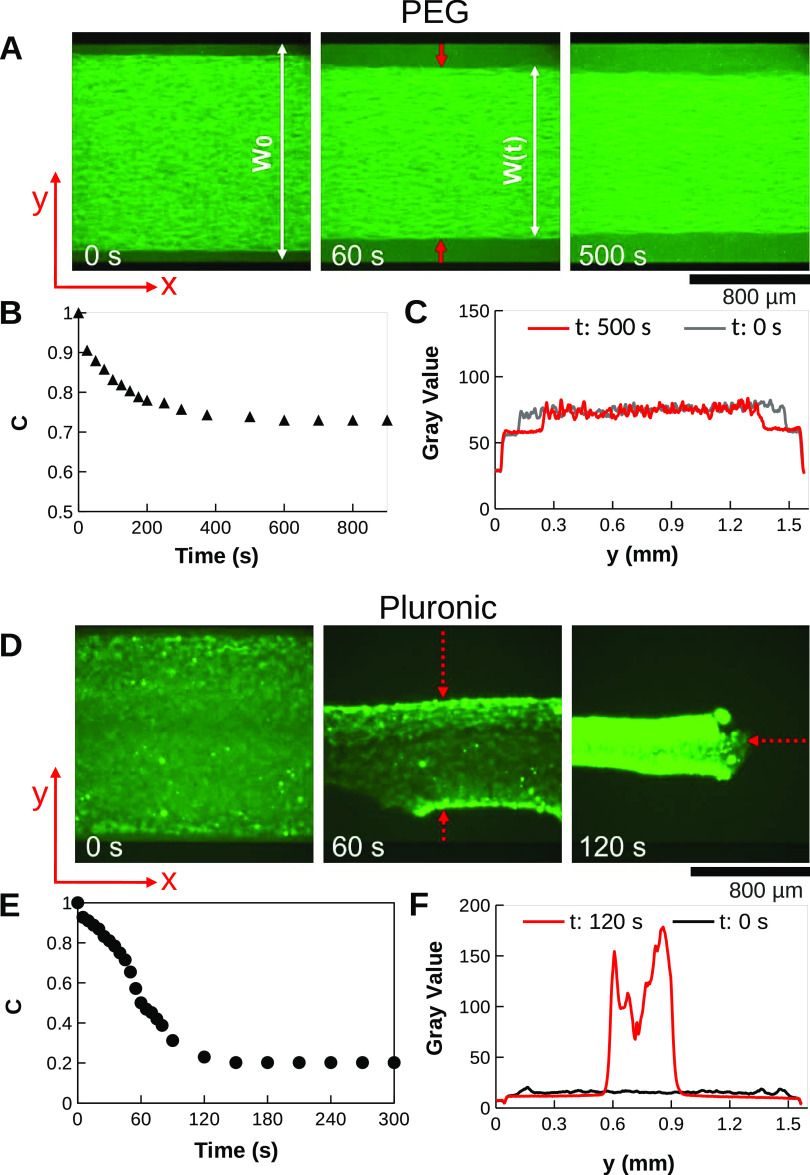
Dynamics of active microtubule networks formed in the presence of kinesin motors and PEG (A–C) or Pluronic (D–F). (A) Contraction of a network with PEG over time. (B) Time dependence of the network width as a fraction of the channel width, *C* = *W*(*t*)/*W*_0_. (C) Density profile across the channel before (gray) and after (red) contraction by motor protein activity. (D) Contraction of an active network with Pluronic over time. (E) Relative network width as a function of time. (F) Density profile across the channel before and after contraction by motor protein activity.

Figure 5A shows the behavior of such active networks. As in the passive case, the depletion attraction resulted in formation of visible bundles that showed a nematic organization. Again, the network had a mesh structure. We quantified the network contraction using its transverse width over time, relative to its initial width, *C* = *W*(*t*)/*W*_0_. The relative width shrank to *C* = 26% in 500 s. After this time, the network reached a plateau, as shown in Figure 5B,C. In our previous study, we observed that network contraction was visible even without the contribution of the motor proteins.^21^ However, it reached a value of 7%, which shows that in the active PEG network the motor proteins contribute most to network contraction.

The active Pluronic network showed a different appearance than PEG already at the onset of the experiment. As in the passive case, it neither showed long bundles nor longitudinal alignment along the channel. The contraction of this networks was stronger and faster compared to the contraction of the PEG one (Figure 5D–F). The system contracted up to 70% within 120 s, and, in some cases, it broke and showed isotropic contraction (Figure 5D).

We explain the difference in the contraction again with the different distribution of the microtubules within the network. In the PEG network, the nematically oriented bundles are subjected to weak active stress. The motor forces almost cancel out in the network and the macroscopic response is due to a small force imbalance as we showed in a previous study.^21^ On the other hand, the isotropic distribution of the bundles in the Pluronic network and the increased cross-linking promote network connectivity. A sufficiently connected network is required for contractility as it can transmit the contractile stresses generated by the motor activity. The unbalanced tension at the edge of the network in contrast to the isotropic tension within the network can also play a role in the contraction and explain the inward movement.^38−40^

Interestingly, the behavior of an active Pluronic network substantially changes by varying microtubules and Pluronic concentrations in solution and motor protein properties. Indeed, a previous study showed that such networks exhibit a nematic order in filament arrangement and contract and wrinkle at a high microtubule concentration when a suitable motor of the kinesin family is used.^41^ However, in the study presented here, the systems had a relatively low volume fraction of microtubules (0.001), and by mixing it with kinesin-1, we did not observe such behavior. For this reason, we focused our investigation on the characterization and comparison of the PEG and Pluronic networks made as described above and are interested in their common contraction behavior.

### Depletion by a Mixture of PEG and Pluronic in Active Networks

**Figure 6:**
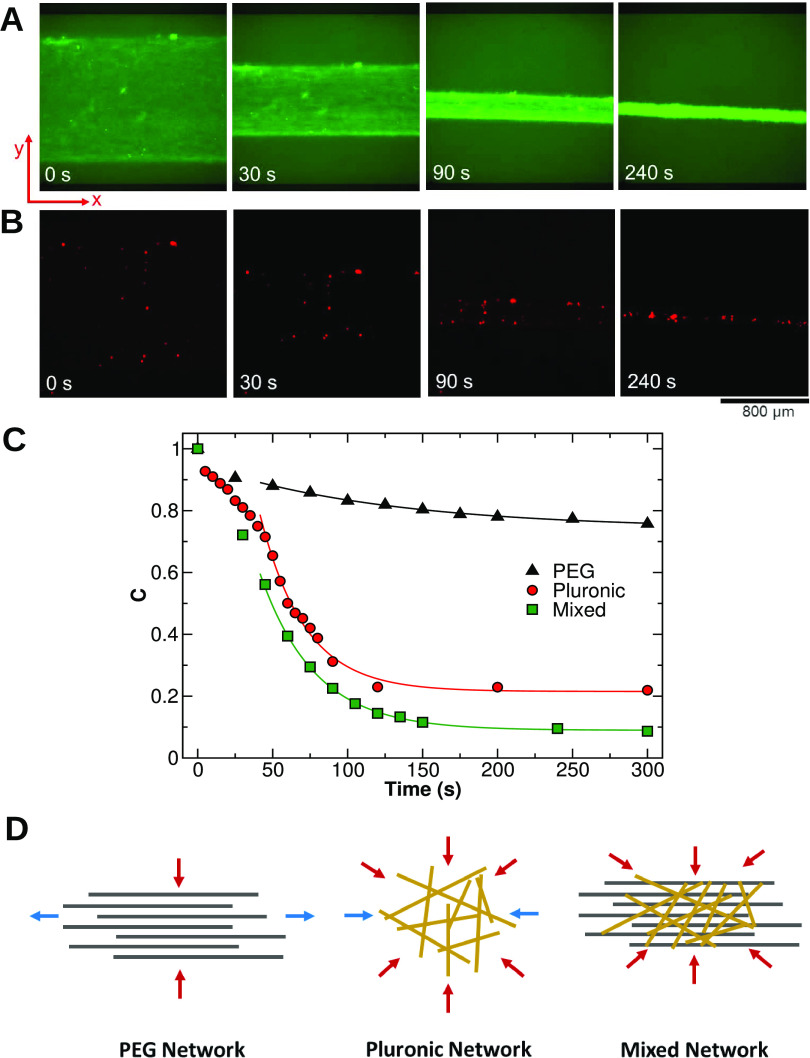
Contraction of an active microtubule network with depletion forces induced by a 1:1 mixture of PEG and Pluronic. (A) Contraction of the microtubule (green) network over time. (B) Distribution of kinesin clusters (red) during contraction. (C) Comparison of network contraction rates with PEG, Pluronic, and mixed depletants. (D) Bundle distribution and active stress in a PEG nematic network, Pluronic isotropic network, and mixed network.

Once it was observed that PEG and Pluronic contributed differently to the contraction of microtubule networks as well as to their final shape and pattern formation, we raised the question about their cooperative contribution to the dynamics of the system. In this regard, a 1:1 ratio mixture of PEG and Pluronic (maintaining the overall 1% concentration of the depletant in the final solution) was tested in active networks. Figure 6 shows the dynamics of network contraction by the mixture of depletants (mixed network). Interestingly, the resulting network showed a mechanical behavior that is a combination of the features of both depletant-induced networks. We could observe long and thick bundles that were longitudinally aligned along the *x*-axis of the channel, as seen in the PEG networks. However, these bundles were embedded in a more compact network, typical for Pluronic. Moreover, the mixed-depletant network contracted faster than the individual single-depletant networks. At the onset of the experiments (Figure 6A), we observed that the mixed network already contracted by 25%. Observing its contraction after 30 s, it is clear that the system was faster than the Pluronic network, which took 60 s for a comparable contraction. The mixed network continued to contract, reaching a plateau at 90% contraction after 240 s without any tear as sometimes observed in the Pluronic network. By labeling the motor proteins, we could observe that the confinement of microtubules to 10% of the width colocalized with local motor protein accumulation (Figure 6B). Figure 6C compares the contraction of the three active cases (PEG, Pluronic, mixed networks). By fitting the final stage of the contraction with an exponential function, we could estimate the time constant τ of the three different active networks. The mixed-depletant network is characterized by τ_mix_ = 36.5 s, the Pluronic network by τ_Pluronic_ = 29.7 s, 4-fold faster than the PEG network, characterized by τ_PEG_ = 123 s.

We explain it with the force distribution that characterizes the networks. As mentioned above, the motor proteins in the nematic PEG network exert both contractile and extensile forces between microtubule pairs. Under this condition, the motor forces in the network almost cancel out and the macroscopic response is due to a small force imbalance as we showed in a previous study.^21^ It results in an extensile stress along the nematic order and contractile in the perpendicular direction, as shown in Figure 6D. On the other hand, the Pluronic network contracted isotropically because of the distribution of the bundles and their increased cross-linking. When the two effects are combined, the contraction forces along the longitudinal axis of the channel due to the Pluronic-induced organization cancel out the extensile forces in the opposite direction due to the PEG-induced arrangement. Thus, the resultant forces act mainly along the transverse axis of the channel without contribution that could break the network (Figure 6D). In this way, a network is created that can contract over time as long as ATP is available to fuel the motors.

This demonstrates how the combined action of the two depletant can be used to tune the features of the final material. The results show that we could obtain new network features by mixing the two depletants as the mixed network contracted as quickly as the Pluronic network and was as robust as the PEG network. The combination of these features improved the properties of the resultant network that could continuously contract into a small volume.

## Conclusions

In this study, we investigated the different depletion effects exerted by the two widely used polymers, PEG and Pluronic, when applied to a diluted solution of rod-like biopolymers such as microtubules. We show that their radius of gyration as well as the tendency to build micelles at a certain concentration drastically modify the structure and dynamics of the biopolymer network they are included into. The bundles in PEG networks display a nematic order, whereas Pluronic networks are characterized by shorter, isotropically arranged bundles.

The isotropic structure of the Pluronic network and its tendency to behave like a deformable sheet are well demonstrated by its wrinkling response to a geometrical obstacle. We confirmed it using a simple theoretical model that shows that the Pluronic network indeed behaves like a 2D elastic plate when subjected to a point force. The different bundle distribution within the network also influences the behavior of the active network. We proved that we can switch between a porous slow contracting network and a compact gel-like fast responding system using either PEG or Pluronic, respectively. Finally, we showed that the combination of the two depletants resulted in a new material with properties common to both individual depletant effects.

These results show the suitability of depletion and the consequent osmotic pressure to tune soft material properties and the potential for engineering new material features by mixing different depletants. This study proposes a simple way to design controllable bioinspired materials. It would be a revolutionary achievement if such biologically derived materials could leave the laboratory and find applications in important fields such as biomedicine due to their biocompatibility.
